# Effect of Coconut Protein and Xanthan Gum, Soybean Polysaccharide and Gelatin Interactions in Oil-Water Interface

**DOI:** 10.3390/molecules27092879

**Published:** 2022-04-30

**Authors:** Yi Yang, Dong Xiang

**Affiliations:** 1 College of Food Science and Engineering, Hainan University, No.58 Renmin Avenue, Haikou 570228, China; yangyi0502@outlook.com; 2 Key Laboratory of Food Nutrition and Functional Food of Hainan Province, No.58 Renmin Avenue, Haikou 570228, China

**Keywords:** coconut protein, hydrocolloid, xanthan gum, soybean polysaccharide, gelatin, interaction, interface, emulsion stabilization

## Abstract

We report on our study of the interactions between coconut protein extracted from coconut meat and three hydrocolloids (gelatin, xanthan gum, and soybean polysaccharide) and their interfacial adsorption and emulsification properties. We used Zeta potential, fluorescence spectroscopy scanning and ITC to investigate the interactions between a fixed concentration (1%) of coconut protein and varying concentrations of hydrocolloid. Through the interfacial tension and interfacial viscoelasticity, the interfacial properties of the hydrocolloid and coconut protein composite solution were explored. The physical stability of the corresponding emulsion is predicted through microstructure and stability analysis. Xanthan gum forms a flocculent complex with coconut protein under acidic conditions. Soy polysaccharides specifically bind to coconut protein. Under acidic conditions, this complex is stabilized through the steric hindrance of soy polysaccharides. Due to gelatin-coconut protein interactions, the isoelectric point of this complex changes. The interfacial tension results show that as time increases, the interfacial tensions of the three composite solutions decrease. The increase in the concentration of xanthan gum makes the interfacial tension decrease first and then increase. The addition of soybean polysaccharides reduces the interfacial tension of coconut protein. The addition of xanthan gum forms a stronger elastic interface film. Emulsion characterization showed that the gelatin-added system showed better stability. However, the addition of xanthan gum caused stratification quickly, and the addition of soybean polysaccharides also led to instability because the addition of polysaccharides led to a decrease in thermodynamic compatibility. This research lays the foundation for future research into coconut milk production technology.

## 1. Introduction

During coconut milk production, different phenomena are observed depending on the hydrocolloid added. Thus, coconut protein appears to show different interactions with different hydrocolloids. Food emulsion is a thermodynamically unstable multiphase system, which is prone to unstable phenomena such as aggregation and flocculation [1]. Therefore, stabilizers (hydrocolloids) are usually added in daily production to increase the stability of oil-in-water emulsions. The researchers used different hydrocolloids to mix with plant protein to increase its emulsifying ability [2], such as HM pectin [3], dextran sulfate [4], carboxymethyl cellulose [5], xanthan gum, and pectin [6]. These results indicate that the type and concentration of polysaccharides have a great influence on the system. In any protein-polysaccharide system, there are protein–polysaccharide interactions [7], and the major non-covalent intermolecular forces between protein and polysaccharide were dominated by electrostatic and hydrophobic interactions, steric exclusion, and hydrogen bonding. In the protein-hydrocolloids system, there are not only protein–polysaccharide interactions but also protein–protein interactions [8].

In recent years, the “green” trend in the pharmaceutical, cosmetic, and food industries has prompted people to have a great research interest in the realization of completely plant-based protein–polysaccharide conjugates [9,10]. Our research is to replace casein with coconut protein, which has emulsifying activity and surface properties and can be used to stabilize oil-in-water emulsions. However, the stabilization behavior of vegetable protein is easily affected by temperature, pH, ionic strength, and other factors, which leads to the instability of emulsions [11,12]. Therefore, we chose three representative hydrocolloids to improve the stability of the coconut protein emulsion. Xanthan gum is free of protein, soy polysaccharide with a small amount of protein, and pure protein hydrocolloid gelatin.

Coconut fruit is comprised of approximately 38.5% shell, 51.7% flesh, and 9.8% water [13]. The composition of coconut meat (the flesh) is 35.2% fat, 3.8% protein, and 40.9% moisture [14]. Although coconut meat has a low protein content, coconut protein recycling remains an economically attractive enterprise because of widescale coconut tree cultivation [15]. Xanthan gum is an extracellular heteropolysaccharide secreted by gram negative bacteria of the genus Xanthomonas [16]. XG is a kind of rigid linear polysaccharide with negative charge [17]. Due to its special rheological properties, it has functional properties such as emulsification, thermal stability, and thickening. It can be used as an emulsifier, stabilizer, and gel enhancer in the food industry. It can resist the Brownian motion of oil droplets and maintain the static stability of the emulsion. This food additive maintains a stable viscosity over a wide range of temperature and pH [18], and xanthan gum is often used as a food thickener and stabilizer [19]. As a non-adsorbent polysaccharide, xanthan gum has no surface activity, and it is usually mixed with emulsifiers (e.g., protein) to improve its interface properties [20]. The effects of protein emulsifier on xanthan gum differ depending on pH. It has been widely used in food emulsions. Soluble soybean polysaccharide (SSPS) is a water-soluble polysaccharide extracted and refined from soybeans. It is mainly composed of dietary fiber from soybean cotyledons. SSPS shows relatively low viscosity and high stability in an aqueous solution. Its structural conformation in an aqueous solution is that of a flexible random coil [21]. SSPS is an anionic polysaccharide, and steric repulsive forces dominate interactions during dispersion [22]. The protein part of SSPS anchors the carbohydrate part at the oil/water interface, and the long hydrophilic chain forms a thick hydration layer of about 30 nm, which can prevent droplets from coalescing through steric repulsion [23], and its emulsion stability is not affected by pH value and ionic strength [24]. Gelatin is formed by the hydrolysis and degradation of collagen under acidic or alkaline conditions. When gelatin solution is cooled below room temperature, a triple helix structure is formed. These triple helices subsequently associate to form a 3D network, with a corresponding increase in gel strength. However, the natural conformation of the protein can only be partially restored. Even after the sample is annealed for several hours or days, a proportion of the sample remains in the random coil conformation. Furthermore, this gel is not in equilibrium. When the temperature rises above approximately 30 °C, a spiral-to-helix reverse transition occurs, and the gel becomes a liquid again [25]. Gelatin is a water-soluble protein macromolecule derived from collagen. It can migrate from the water phase to the oil/water interface, forming a thin molecular layer to stabilize the emulsion [26].

Moreover, the structure of the protein is determined both by interactions with polysaccharides and by interaction of the protein with itself. In a previous study, Elmer et al. [27] demonstrated that the tertiary structure of a protein changes after addition of polysaccharide, and that biopolymers interact through electrostatic attraction. However, to the best of our knowledge, there are still gaps in the research on the interaction between coconut protein and hydrocolloids. The type and concentration of the hydrocolloid have a great influence. The protein content of the three hydrocolloids increased sequentially in our study (xanthan gum < soybean polysaccharide < gelatin). The purpose of the present study is to explore the interaction between coconut protein and three different hydrocolloids, to characterize the interaction by means of particle size analyzers and ITC, and to study three different hydrocolloid and coconut protein stabilized emulsions, the interfacial adsorption properties of the oil–water interface of the system (interfacial pressure and interfacial swelling viscoelastic properties) and the mixture of hydrocolloids and coconut protein stable emulsion properties (microstructure, flow behavior and stability). In addition, the relationship between the interaction of hydrocolloids and coconut protein in the oil–water interface and their emulsion properties was revealed, and the mixture of hydrocolloid and coconut protein is used as a functional ingredient in the food system.

## 2. Results and Discussion

### 2.1. State Diagrams

**Figure 1:**
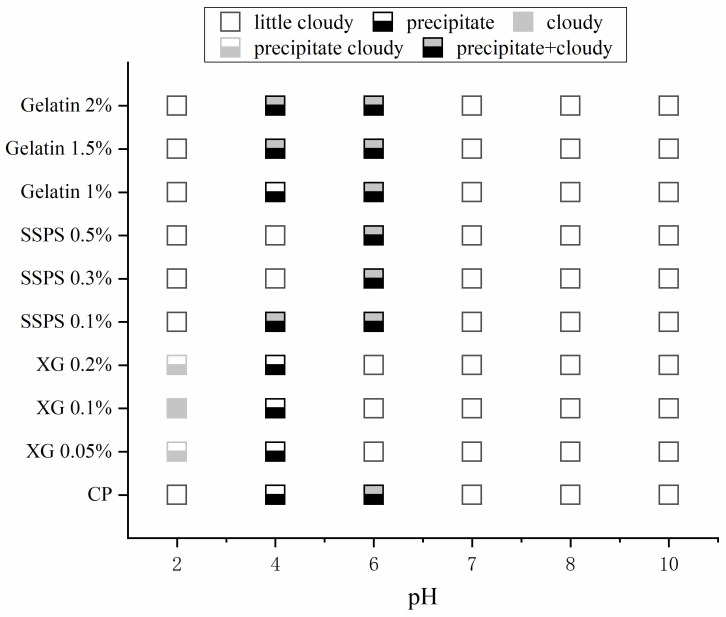
State diagrams of CP-Hydrocolloid systems at different pH values and different concentration of the three hydrocolloids (CP-Coconut protein, XG-Xanthan gum, SSPS-Soluble soybean polysaccharide).

To explore the interaction between CP and hydrocolloid (XG, Gelatin, and SSPS), the phase behavior of the complex system was observed [28]. The state diagrams for the complex system under different pH and hydrocolloid concentration conditions are shown in Figure 1. The state diagrams reveal that the CP exhibits different phase behaviors when compounded with different hydrocolloids. Likewise, the concentration of hydrocolloids affected phase behavior.

In the range pH 7–pH 10, all complex solutions appeared homogeneous. At pH 6, the CP-XG complex solution still demonstrated a uniform milky white phase. However, the other complex solutions demonstrated the same behavior as CP (lower layer, precipitated protein; upper layer, a turbid solution). Thus, no protein precipitate was observed in the CP-XG complex solution at pH 6. One possibility is that the precipitated protein particles are uniformly fixed in the solution because of the viscosity of XG [29]. Another possibility is that the interaction between XG and CP at pH 6 precludes large-scale protein precipitation.

At pH 4 (which is close to the isoelectric point of coconut protein), a large amount of coconut protein is precipitated in the CP solution, and the supernatant liquid was correspondingly clear [30]. In the CP-XG complex solutions, floccules can be observed. Since irreversible floccules are produced under acidic conditions, the observed precipitate results from the flocculation and precipitation of coconut protein [28]. In the CP-Gelatin complex solutions (pH 4), less precipitate was observed, indicating that the combination of CP and Gelatin may change the isoelectric point of the complex. In the CP-SSPS complex solutions (pH 4), some precipitate was observed at low concentrations of SSPS compound solution. However, the amount of precipitate was significantly reduced (when compared with the other groups of compound solution). At higher concentrations of SSPS, the CP-SSPS complex solutions exhibited a uniform milky white phase. Thus, under acidic conditions, the soybean polysaccharide interacts with CP making the composite liquid stable. As a consequence, coconut protein does not precipitate near the isoelectric point in this system.

At pH 2, most of the complex solutions demonstrated a single homogeneous phase [31]. However, irreversible floccules were observed in the CP-XG complex solution. Thus, CP-XG complex solutions demonstrated phase separation (lower layer, a precipitate of floccules; upper layer, a clear liquid).

### 2.2. Zeta Potential

**Figure 2:**
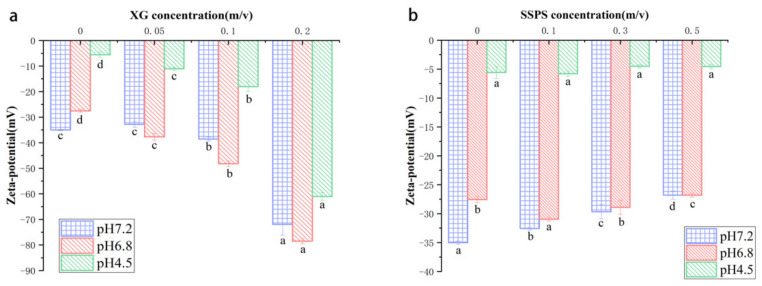

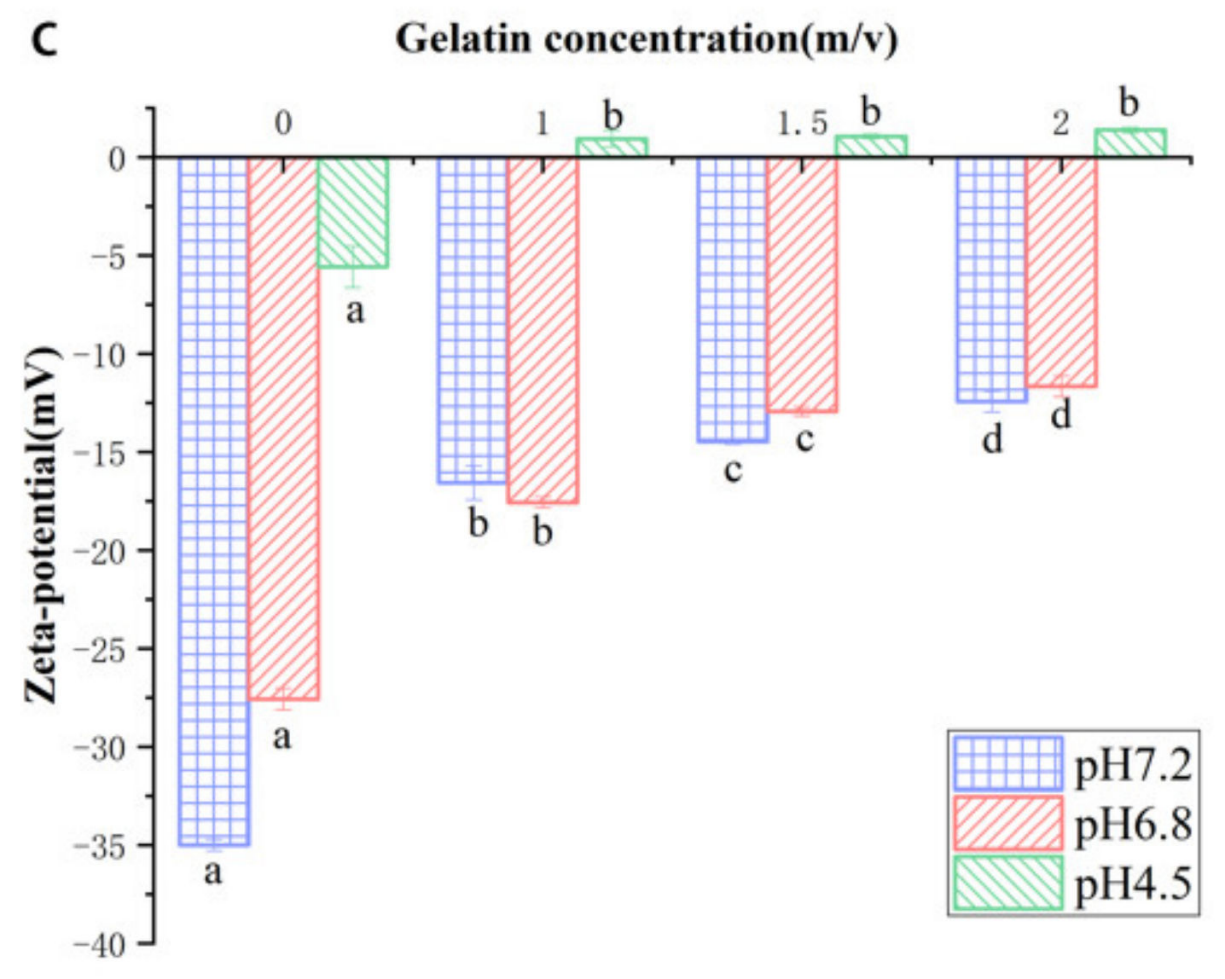
The zeta potential of CP-Hydrocolloid systems at different pH values and different concentrations of the three hydrocolloids. The CP concentration was 1% (*w*/*v*). Letters a–d indicate significant difference in different pH (*p* < 0.05). (**a**) CP-XG; (**b**) CP-SPSS; (**c**) CP-Gelatin. (CP-Coconut protein, XG-Xanthan gum, SSPS-Soluble soybean polysaccharide).

The interaction force and stability of the CP-hydrocolloid complex system can be analyzed through measurement of the Zeta potential. As shown in Figure 2, the potential of the complex system at three specific pH values was measured. As the pH increased, the potential of coconut protein dropped from −5.5 mV to −35 mV.

The potential of the complex system also changed with the addition of (and change in the concentration of) hydrocolloid. The potential of the CP-XG complex solution decreased with an increase in the concentration of XG. This is likely due to an electrostatic attraction between the negatively charged XG and positively charged fragments on the CP molecule [32]. The potential of the CP-SSPS complex solution did not change significantly with SSPS concentration. The potential of the CP-Gelatin complex solution increased with an increase in the concentration of gelatin. Moreover, the absolute value of the potential decreased significantly with gelatin concentration. Thus, the gelatin and CP interact, and this interaction causes the protein in the system to aggregate, making the protein less stable [33].

### 2.3. ITC

**Figure 3:**
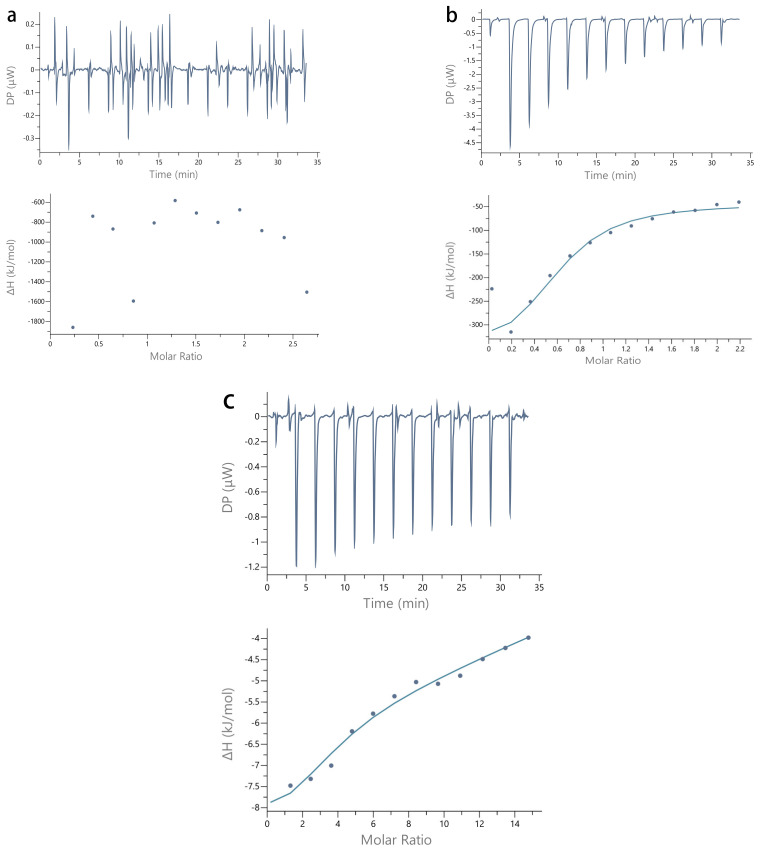
ITC titration curves of CP and hydrocolloids. (**a**) CP-XG; (**b**) CP-SPSS; and (**c**) CP-Gelatin. (CP-Coconut protein, XG-Xanthan gum, SSPS-Soluble soybean polysaccharide).

**Figure 4:**
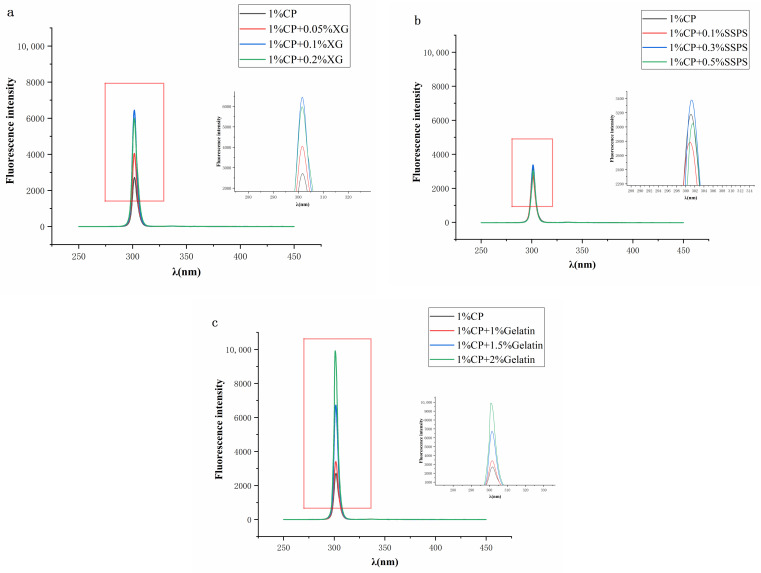
Intrinsic fluorescence intensity of CP-Hydrocolloid systems. (**a**) CP-XG; (**b**) CP-SPSS; (**c**) CP-Gelatin. (CP-Coconut protein, XG-Xanthan gum, SSPS-Soluble soybean polysaccharide).

ITC is widely used to analyze protein–polysaccharide interactions [34,35,36]. The isothermal titration curves of the different hydrocolloids and CP are shown in Figure 3. Titrations of the CP-Gelatin and CP-SSPS systems both demonstrate obvious exothermic peaks. Moreover, the titration curves reveal a similar overall trend. As the number of titrations increases, the exothermic peaks gradually weaken. However, the exothermic heat in each system differs, reflecting the different hydrocolloid interactions with CP. In contrast, titration of CP-XG did not demonstrate regular peaks. As shown in Figure 3 and Figure 4, the titration peaks for CP-XG were very weak, suggesting that there is no specific binding between CP and XG. Furthermore, the titration curve did not conform to a typical ITC curve.

The data for the CP-Gelatin and CP-SSPS systems could both be fit to a model. Fitting their isothermal titration curves yielded the specific binding parameters shown in Table 1. The results reveal that the binding ratio (N) and the binding constant (K) for the CP-Gelatin system are greater (compared with those of the CP-SSPS system). These results reveal that gelatin and coconut protein have a stronger binding capacity. No suitable fitting formula was available to fit the CP-XG ITC curve.

### 2.4. Fluorescence Spectroscopy

Changes to the tryptophan microenvironment have a significant impact on the endogenous fluorescence of a protein. Hence, endogenous fluorescence is often used to detect changes in the spatial structure of proteins. Any increase or decrease in fluorescence intensity indicates an interaction change. In addition, when the solvent exposure of the tryptophan chromophore increases, the maximum fluorescence emission shifts to a longer wavelength (a ‘red shift’). As a corollary, when the solvent exposure of the tryptophan chromophore decreases (i.e., the chromophore is more buried), the maximum fluorescence emission shifts to a shorter wavelength (a ‘blue shift’) [37].

The fluorescence scanning spectrums of the CP-hydrocolloid composite solutions are shown in Figure 4. The λ_max_ value of CP-XG (301.5 nm) did not change with XG concentration (within our experimental concentration range). The λ_max_ value of CP-SSPS demonstrated a slight red shift with SSPS concentration. Thus, the conformation of CP changed in the presence of SSPS, with the tryptophan residues moving to a more hydrophilic environment. The λ_max_ value of the CP-Gelatin composite solution did not change with gelatin concentration (within our experimental concentration range).

### 2.5. SEM

**Figure 5:**
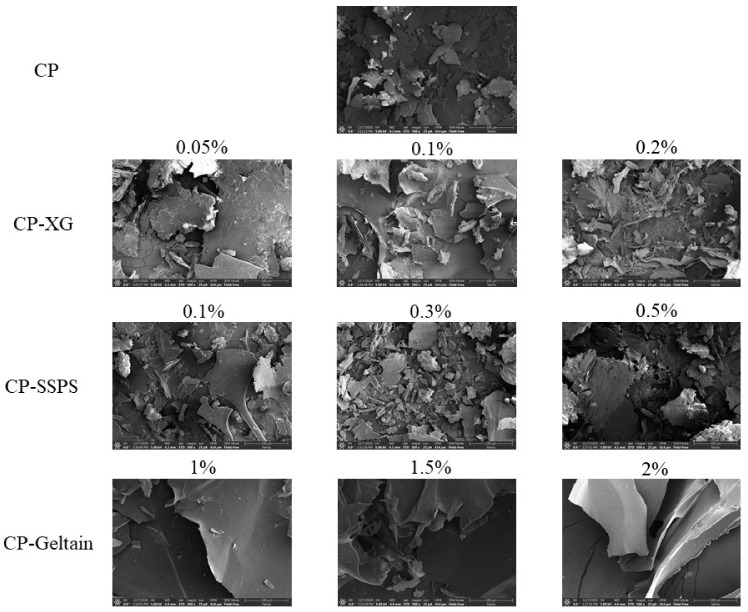
SEM images of CP-Hydrocolloid systems. (CP-Coconut protein, XG-Xanthan gum, SSPS-Soluble soybean polysaccharide).

Scanning electron microscopy was used to observe the microscopic morphology of CP and CP-hydrocolloid complexes (Figure 5). The addition of different hydrocolloids had a significant impact on the microstructure of the CP-hydrocolloid complex. In the absence of hydrocolloid, CP demonstrated a bulky and loose structure. In contrast, CP-XG complex demonstrated a denser structure with smaller pores. The CP-Gelatin complex demonstrated a smooth surface and a large complete block structure. Finally, the CP-SSPS complex demonstrated a loose and uneven structure.

### 2.6. Interfacial Tension

**Figure 6:**
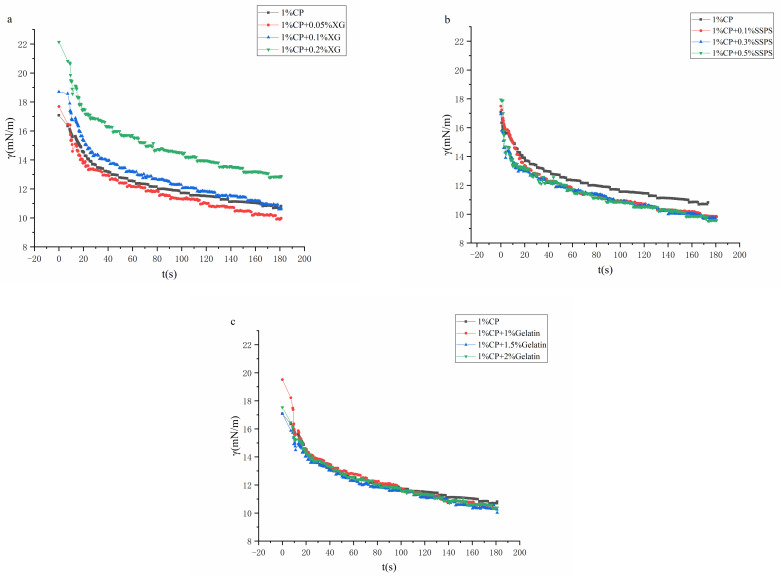
Dynamic interfacial tension (γ) for sample solutions at the oil–water interface. (**a**) CP-XG; (**b**) CP-SPSS; and (**c**) CP-Gelatin. (CP-Coconut protein, XG-Xanthan gum, SSPS-Soluble soybean polysaccharide).

The influence of different hydrocolloids on the adsorption of coconut protein on the oil–water interface is shown in Figure 6. First, when t = 0, the γ value is less than that of pure water, indicating that the system has good surface activity. As time goes by, all samples show a trend of decreasing interfacial tension. In the early stage of adsorption, the interfacial tension decreases rapidly after the mixture is dripped into the oil, and the rate of decrease in the late stage of adsorption is relatively slow [38].

It can be seen from the figure that the interfacial tension of the mixed system of different hydrocolloids and coconut protein is also different. In the XG system, as the concentration of XG increases, the interfacial tension tends to decrease first and then increase. Adding a low concentration of XG will increase the incompatibility of the system, change the structure of the protein, and expose the hydrophobic group [32,39], which is conducive to the interaction between the protein and the oil phase. When the concentration of XG increases, the interfacial tension of the composite system gradually increases, and the emulsification ability gradually decreases. It may be because the XG has a certain hydrophobic interaction with coconut protein, which reduces the exposure of coconut protein hydrophobic groups in water, thereby reducing the interfacial activity. It may also be that XG increases the viscosity of the solution and hinders the adsorption of the system. The addition of soybean polysaccharides reduces the interfacial tension of the system [40], but it is not sensitive to the concentration of soybean polysaccharides. Soy polysaccharides also increase the incompatibility and reduce the interfacial tension. The interfacial tension of the system with gelatin does not change significantly because gelatin is a protein and does not affect the incompatibility [41]. In this experiment, none of the three systems reached a complete adsorption equilibrium. This is because the adsorption process of protein and other macromolecular surface-active substances on the interface is slow [42].

### 2.7. Interfacial Dilatational Viscoelastic Properties

**Figure 7:**
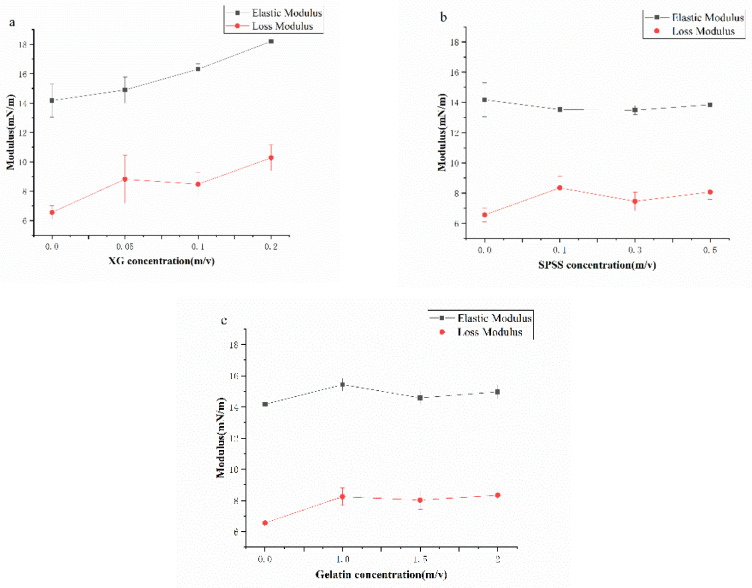
Elastic Modulus and Loss Modulus for samples at the oil–water interface. (**a**) CP-XG; (**b**) CP-SPSS; and (**c**) CP-Gelatin. (CP-Coconut protein, XG-Xanthan gum, SSPS-Soluble soybean polysaccharide).

The elastic modulus and viscous modulus of the interface expansion rheological properties are shown in the Figure 7. It can be seen that the value of the elastic modulus is much larger than the viscous modulus, indicating that the coconut protein interface films is mainly elastic. As the polysaccharide concentration increases, the elastic modulus increases, and the elastic characteristics of the interface films also increase. The phenomenon of adding XG is more significant, which shows that the addition of XG increases the viscoelasticity of the coconut protein interface films [43], and the protein value adsorbed on the oil/water interface is mainly elastic in the interface films structure. Because XG is an anionic non-adsorbent polysaccharide, it will not compete with the protein on the interface. The changes in this system indicate that XG participates in the formation and development of the interface film, which may be due to the electrostatic interaction between macromolecules increasing the expansion modulus of the interface film.

XG significantly improves the viscoelasticity of the oil–water interface film [44], while soybean polysaccharides and gelatin have a smaller effect than XG (the effect on the interface pressure is also small). The effect on the viscoelastic modulus of the interface expansion is relatively small, which may be because the electrostatic effect is less than that of XG, which has better thermodynamic compatibility. When the concentration of hydrocolloid increases, there is not enough space for protein molecules to expand on the interface; the structure is compressed, and the interaction between molecules is inhibited. At high concentrations, the strength of the interface membrane does not change significantly. As the concentration increases, the thermodynamic compatibility between macromolecules decreases, and the repulsion effect increases the amount of protein adsorption, increases the protein density on the interface, increases the probability of interaction between protein molecules, and enhances the network structure of the interface.

### 2.8. Visual Observations

**Figure 8:**
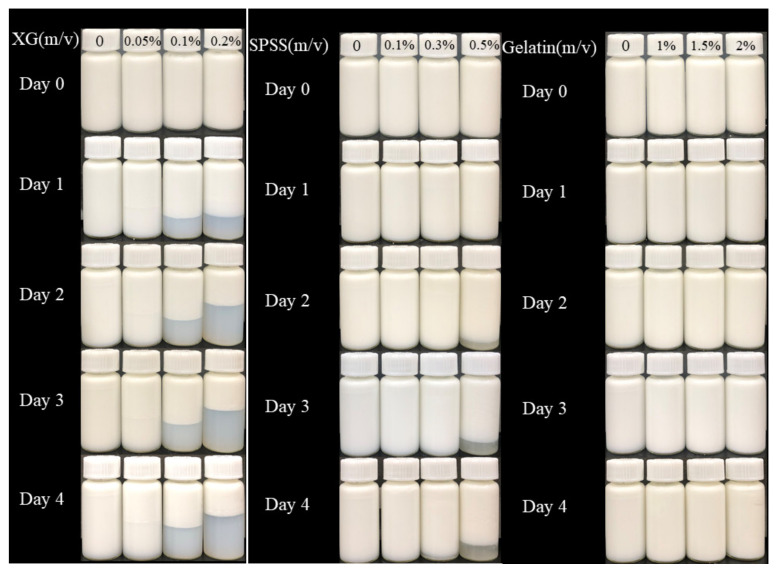
Visual observations of emulsion samples. (CP-Coconut protein, XG-Xanthan gum, SSPS-Soluble soybean polysaccharide).

Figure 8 shows the storage images of three emulsion systems at 25 °C. It can be seen directly from the figure that the storage stability of the emulsion with XG is decreased, while the stability of the coconut protein emulsion without XG is better. After adding a high concentration of XG, serious delamination occurred after 1 day, forming a transparent lower layer. This shows that as the concentration of XG increases, the content of unadsorbed XG in the bulk increases, and the resulting osmotic pressure reduces the distance between droplets until they aggregate, thereby forming the XG repelling area, repelling flocculation [20,45], and the emulsion is seriously stratified. Similar to XG, the addition of SSPS also affects the stability of the emulsion system to a certain extent, but the effect is small. It can be seen that the addition of high-concentration SSPS on the second day caused slight layering of the emulsion, and the system also experienced repelling flocculation [46]. It can be observed that after gelatin is added, the emulsion basically has no stratification phenomenon, so the addition of gelatin enables the system to be stored stably, indicating that the gelatin and coconut protein system can completely emulsify 10% of coconut oil.

### 2.9. Storage Stability

**Figure 9:**
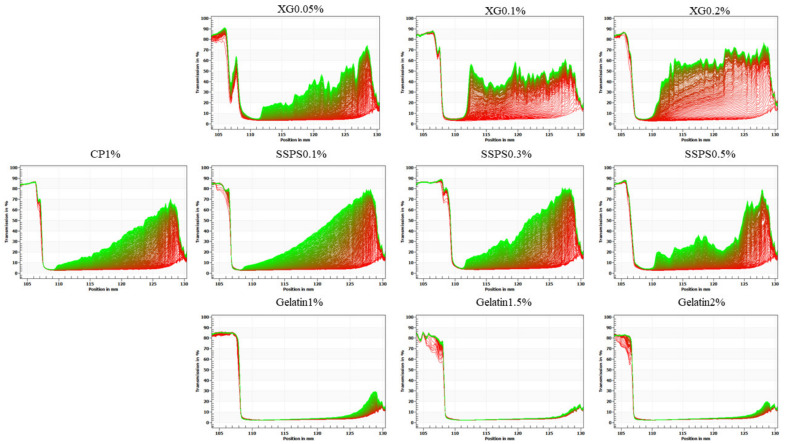
Evolution of transmission profiles of emulsion samples at different concentrations (CP concentration, 1%). All transmission profiles were measured using the LUMiSizer. (**left**: start, red line; **right**: end, green line) (CP-Coconut protein, XG-Xanthan gum, SSPS-Soluble soybean polysaccharide).

Figure 9 shows the light transmittance graph after the stability test. These lines represent the stability image obtained under the conditions of 4000 rpm centrifugation from the 0 to 2.5 h. It can be seen from Figure 3 and Figure 9 that the addition of XG reduces the stability of the emulsion, which is because the addition of XG causes flocculation in the emulsion system. When a low concentration of XG is added, the system has a bridging effect, forming bridging flocculation [47], and the addition of high concentration of XG makes the system form a repulsive flocculation phenomenon [48], and the system is stratified. The effect of SSPS on the emulsion is not as significant as that of XG. The addition of high concentration of SSPS can improve the stability of the emulsion, which is related to the small amount of protein in the soybean polysaccharide. The addition of gelatin can greatly improve the stability of the system. Gelatin is a protein and coconut protein can work together to make the emulsion system stable.

### 2.10. Microstructure of the Oil Droplets in Emulsions

**Figure 10:**
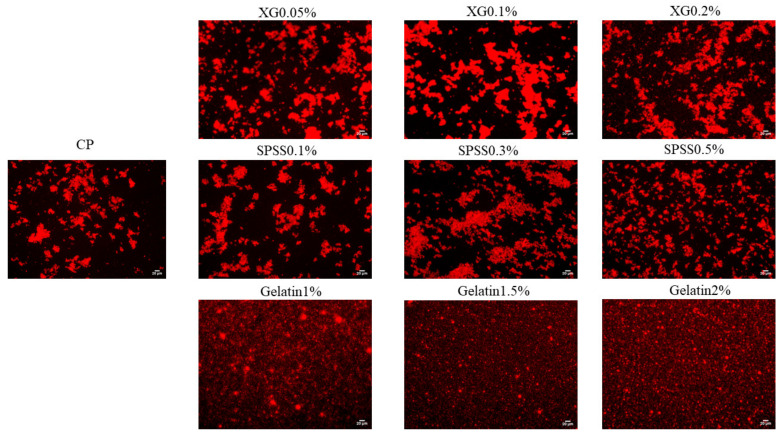
Effect of XG, SSPS and gelatin on the microstructure of emulsions samples. (CP-Coconut protein, XG-Xanthan gum, SSPS-Soluble soybean polysaccharide).

Figure 10 shows the effect of XG, SSPS, and gelatin on the microstructure of coconut protein emulsion. It can be seen that the effects of different hydrocolloids on emulsions are significantly different. When no hydrocolloid is added, there is a small amount of fat globule aggregation, indicating that 1% coconut protein cannot emulsify 10% coconut oil more completely. After adding XG, it can be seen that the fat globules have seriously aggregated, and the emulsion has flocculated [49]. The emulsion with SSPS also showed the same aggregation, but the degree of aggregation was lighter than the system with XG. It shows that the CP-XG emulsion system has a greater degree of flocculation than the CP-SSPS, which also indicates that the thermodynamic compatibility of CP-XG is poor. In the emulsion system with gelatin added, it can be clearly seen that the structure and particle size of the fat globules in the emulsion are uniform. Adding a certain concentration of gelatin can completely emulsify 10% of coconut oil.

## 3. Discussion

**Figure 11:**
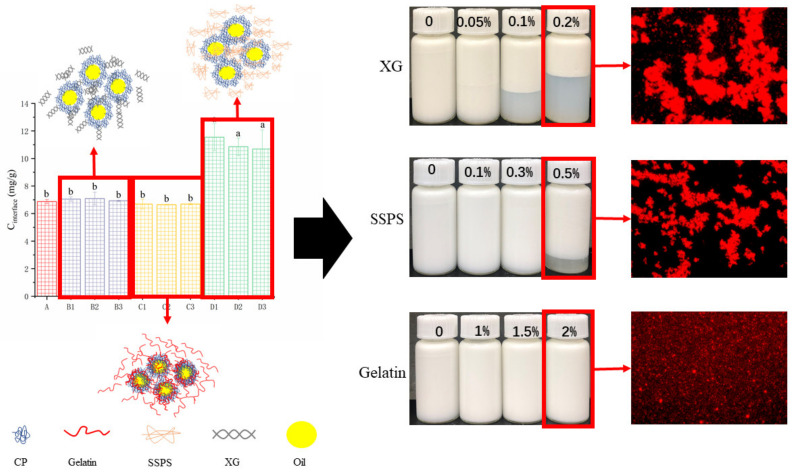
The different emulsification mechanisms of CP-Hydrocolloid systems. (CP—Coconut protein, XG—Xanthan gum, SSPS—Soluble soybean polysaccharide, A—CP, B—“CP-XG”, C—”CP-SSPS”, D—“CP-Gelatin”, “1–3” represents the concentration increase). Letters a, b indicate significant difference in different systems (*p* < 0.05).

The emulsification mechanism of three different hydrocolloids and coconut protein is shown in Figure 11. The high concentration of XG can enhance the elastic film at the oil–water interface during the emulsification process, but due to the strong electrostatic effect and repulsive flocculation, the emulsion has obvious layering. The emulsion emulsified by SSPS also exhibited the phenomenon of repelling flocculation and thermodynamic incompatibility. However, the repelling effect is weaker than that of emulsions containing XG. From the concentration of the interface protein, the system with added polysaccharides did not replace the interface protein, which remained the same as before it was added. The emulsion system added with gelatin significantly increases the concentration of the interface protein. Coconut protein and gelatin stabilize the emulsion together without thermodynamic incompatibility, so the stable emulsion was obtained.

## 4. Materials and Methods

### 4.1. Coconut Materials and Chemicals

The coconut was obtained from a local supermarket (Hainan, China). All other chemicals used in this study were of analytical grade unless otherwise specified.

### 4.2. Extraction of Coconut Protein

Fresh coconut meat stem was cut into small pieces and placed in a crusher. The coconut residue was then filtered to obtain coconut milk. Coconut milk and phosphate buffer (pH 8) were mixed at a material-to-liquid ratio of 1:4 mL/mL. The suspension was thoroughly mixed and then incubated at 40 °C for 3 h. Next, the mixture was centrifuged (8000× *g*) for 10 min, and the supernatant was collected. The pH of the supernatant was adjusted to pH 4.5, and the protein was allowed to precipitate for 1 h. To obtain crude protein, the sample was first centrifuged at 16,000× *g* for 10 min, and the resulting precipitate was then washed in neutralizing buffer. The crude protein was then dialyzed (membrane cut-off, 3500 kDa) at 4 °C for 2 d. Finally, the dialyzed sample was freeze-dried to obtain the desired coconut protein (CP).

### 4.3. Preparation of Coconut Protein and Hydrocolloid Complex Solution

Weighed amounts of CP, Gelatin (purchased from Sigma-Aldrich, St. Louis, MI, USA), XG (purchased from Sigma-Aldrich), and SSPS (Soyafibe-S-CA100; crude protein 6.3%, moisture 5.6%, ash 7.4%) were separately dissolved in deionized water at room temperature. The final concentration of each solution was: CP solution, 2% (*w*/*v*); gelatin solution, 4% (*w*/*v*); XG solution, 0.04% (*w*/*v*); and SSPS solution, 1% (*w*/*v*). To ensure full hydration, the protein solution was incubated at 4 °C for 12 h. The CP solution was then combined with hydrocolloid solutions at room temperature (in fixed ratios) to prepare the coconut protein-hydrocolloid complex solutions. After the pH was adjusted to neutral, the solutions were thoroughly mixed.

### 4.4. Emulsions Preparation

The oil-in-water emulsions, containing 90% complex mixtures solutions and 10% coconut oil, were prepared firstly by a high-speed homogenizer operated at 10,000 rpm for 1 min and then immediately homogenized with a high-pressure homogenizer at 40 MPa for once. Sodium azide (0.02%) was added as an anti-microbiological agent. The emulsions were stored at room temperature for further analysis. All tests were completed within 24 h.

### 4.5. Phase Diagram of CP-Hydrocolloid Complex Solution

The pH of the CP-hydrocolloid complex solutions prepared in 2.3 were adjusted to set pH values (pH 2–pH 10) using NaOH and HCl. After thorough mixing, the solutions were incubated at room temperature (25 °C) for 12 h, and the state of each solution was observed.

### 4.6. Phase Diagram of Emulsions

The prepared emulsion was poured into a 10 mL sample bottle, the solutions were incubated at room temperature (25 °C) for 0, 24, 48, and 72 h and the state of each emulsion was observed.

### 4.7. Zeta Potential Measurement

ZS Zetasizer Nano (Malvern Panalytical Ltd., Malvern, UK) was used to measure the zeta potential of the composite solutions. The instrument determined the zeta potential with the Henry equation [50]. The measurement conditions were as follows: measurement temperature, 25 °C; equilibration time, 2 min. Each sample was measured three times, and the average value was reported.

### 4.8. Isothermal Titration Calorimetry (ITC)

A Malvern MICROCAL PEAQ-ITC was used for all ITC experiments. The experimental conditions were standardized as follows: sample pool volume, 200 μL; injection needle volume, 40 μL; injection volume, 3 μL; total number of titrations, 13; injection time, 6 s; titration interval, 150 s; and stirring rate, 750 rpm. While the sample cell contained a low-concentration solution, the injection needle contained a high-concentration solution. The protein and hydrocolloid solutions were fully dialyzed before each experiment to avoid experimental interference from ionic strength or pH. The following thermodynamic parameters were calculated from the measured isotherms by curve fitting: binding stoichiometry (N); binding constant (K); enthalpy (ΔH); and entropy (ΔS).

### 4.9. Fluorescence Spectroscopy

An F-280 fluorescence spectrophotometer (Tianjin Gangdong Sci. & Tech. Co., Ltd., Tianjin, China) was used to measure the fluorescence spectra of the protein and hydrocolloid solutions. The endogenous fluorescence of coconut protein was measured after a 1000-fold dilution of the composite solutions. The experimental parameters were: excitation, 295 nm; spectrum collection range, 250–450 nm; scanning speed, 12,000 nm/min; excitation and emission slits, 5 nm. The experimental value reported was the average of three scans.

### 4.10. SEM

The coconut protein-hydrocolloid complex solutions were first freeze-dried. The freeze-dried samples were then transferred to a black conductive glue containing the adhesive for fixation, and sprayed with gold. The microscopic morphology of these samples was observed using a Thermo Scientific Verios G4 UC field emission scanning electron microscope (Thermo Fisher Scientific Inc., Waltham, MA, USA). The magnification used was 500 times.

### 4.11. Interfacial Adsorption Characterization

The interfacial adsorption property of complex mixtures at the oil–water interface was measured by an optical contact angle meter with oscillating drop accessory and was assessed by the interfacial tension (γ), adsorption kinetics, and interfacial dilatational viscoelastic properties. All experiments were carried out at 25 °C.

#### 4.11.1. Interfacial Tension Measurements

The interfacial tension (γ) was determined using OSA 100. The complex mixtures were placed in the syringe. Then a drop of sample solution was delivered into an optical glass cuvette containing coconut oil. The image of the drop was continuously taken from a Charged Coupled Device (CCD) camera (Ningbo NB Scientific Instruments Co., Ltd., Ningbo, China) and digitized. Interfacial tension is defined as γ; γ is the time-dependent interfacial tension of complex mixtures.

#### 4.11.2. Adsorption Kinetics and Interfacial Dilatational Viscoelastic Properties Analysis

The adsorption of kinetic and interfacial dilatational viscoelastic properties of complex mixtures at the oil–water interface was performed according to the method described by Liu et al. (2011). The interfacial dilatational viscoelastic properties, including dilatational modulus (E), dilatational elasticity (E_d_), dilatational viscosity (E_v_), and loss-angle tangent (tanθ, tanθ = E_d_/E_v_), were measured as a function of adsorption time at deformation amplitude of 10% and at frequency of 0.1 Hz and analyzed by SurfaceMeter™ software (Ningbo NB Scientific Instruments Co., Ltd., Ningbo, China) automatically.

### 4.12. Fluorescence Microscope Analysis

A volume of 1 mL of emulsions were put into tubes, and 40 μL of fluorescent dyes (0.1% Nile red and 1% Nile blue) 1,2-propanediol solution (2% water) was added into the tubes. Emulsions were vortexed totally. Then, 3 μL of emulsions were added onto glass microslides, and square cover glasses were placed onto the emulsions to prepare samples.

### 4.13. Stability of Complex Solution

A LUMiSizer dispersion analyzer (LUMiSizer-651 12 channel, Berlin, Germany) was used to analyze the stability of each emulsion at 25 °C (wavelength of detection light, 850 nm; light factor, 1.00). The test was performed at 4000 rpm (RCA, 1878.24 g) for 2.5 h. The total number of contour lines was 300. The instability index was calculated from the meniscus to the bottom of the complex solution.

### 4.14. Statistics Analysis

Data were analyzed using SPSS (version 22.0, IBM SPSS Statistics, IBM Corporation, Armonk, NY, USA). All experimental values were measured in triplicate and expressed as mean ± standard deviation. All significant differences were identified using Duncan’s multiple range test (*p* < 0.05).

## 5. Conclusions

In this work, we studied the interaction of three different hydrocolloids and coconut protein and their influence on the coconut oil emulsion system. First, the results of phase diagram and Zeta potential analyses reveal that CP-XG complexes are formed through electrostatic interactions and hydrophobic interactions. The results of ITC reveal that a specific binding interaction is present in both the CP-SSPS and CP-Gelatin complexes. The concentration of XG in the mixture had a large impact on the electrostatic interactions. The presence of gelatin also affected electrostatic interactions, although this effect was not concentration-dependent. In contrast, SSPS had no significant effect. Due to its higher viscosity, XG stabilized the mixture system. Then, we explored the oil–water interface properties of the composite solution of three hydrocolloids and CP. We found that the addition of XG significantly changed the interface adsorption performance of the composite solution, forming a stronger elastic interface film, which has a significant relationship with the concentration. The addition of SSPS also reduces the interfacial tension of the system, but it is not sensitive to concentration. The addition of gelatin did not significantly change the interface characteristics of the system. Lastly, we prepared the corresponding emulsion for the above composite solution and compared three emulsions stabilized by hydrocolloid and coconut protein complex; CP-gelatin stabilized emulsion showed higher stability and lower fat globule size. XG and SSPS enhance the interface properties of coconut protein, but the emulsion stability of the composite solution is reduced due to flocculation. When discussing the emulsion stability of these three composite solutions, static electricity and steric effects should be considered at the same time, so as to improve the appearance of flocculation and obtain long-term stable emulsions. The results of this study will help us understand the interaction of the three hydrocolloids on coconut protein, as well as the effects of interfacial properties and emulsifying properties. It provides a reference for the production and processing of green coconut protein beverages.

## Figures and Tables

**Table 1:** Binding parameters for CP and hydrocolloids.

	N	K (M)	ΔH (KJ/mol)	ΔG (KJ/mol)	TΔS (KJ/mol)
**SSPS**	0.588	9.79 × 10^−9^	−355	−34.3	301
	**N_1_/N_2_**	**K_1_** **(M)**	Δ**H_1_** **(KJ/mol)**	**K_2_** **(M)**	Δ**H_2_** **(KJ/mol)**
**Gelatin**	2.69/1.51	4.33 × 10^−6^	−11.5	136 × 10^−6^	−6.34
